# An Observational Report of Screen Time Use Among Young Adults (Ages 18-28 Years) During the COVID-19 Pandemic and Correlations With Mental Health and Wellness: International, Online, Cross-sectional Study

**DOI:** 10.2196/38370

**Published:** 2022-08-24

**Authors:** Michelle Teresa Wiciak, Omar Shazley, Daphne Santhosh

**Affiliations:** 1 Department of Microbiology Saint James School of Medicine Saint Vincent and the Grenadines Campus Arnos Vale Saint Vincent and the Grenadines

**Keywords:** coronavirus, COVID-19, pandemic, mental health, depression, anxiety, screen-time usage, young adults, students, international study, observational study, cross-sectional study, smoking

## Abstract

**Background:**

Screen time (ST) drastically increased during the COVID-19 pandemic, but there is little research on the specific type of ST use, degree of change from before COVID-19, and possible associations with other factors. Young adults are a particular interest since previous studies have shown the detriment ST has on a young person’s health. With the combination of a life-changing pandemic, there are unreached depths regarding ST and young adults. This study aims to provide insight into these unknowns.

**Objective:**

This study aims to assess ST in 3 domains (entertainment, social media [SM], and educational/professional) in young adults early in the COVID-19 pandemic; identify trends; and identify any correlations with demographics, mental health, substance abuse, and overall wellness.

**Methods:**

An online, cross-sectional observational study was performed from September 2020 to January 2021 with 183 eligible respondents. Data were collected on ST, trauma from COVID-19, anxiety, depression, substance use, BMI, and sleep.

**Results:**

The average total ST during COVID-19 was 23.26 hours/week, entertainment ST was 7.98 hours/week, SM ST was 6.79 hours/week, and ST for educational or professional purposes was 8.49 hours/week. For all categories, the average ST during COVID-19 was higher than before COVID-19 (*P*<.001). We found ST differences between genders, student status, and continent of location. Increased well-being scores during COVID-19 were correlated with greater change in total ST (*P*=.01). Poorer sleep quality (*P*=.01) and longer sleep duration (*P*=.03) were associated with a greater change in entertainment ST (*P*=.01). More severe depression and more severe anxiety was associated with the amount of entertainment ST (*P*=.047, *P*=.03, respectively) and greater percent change in SM (*P*=.007, *P*=.002, respectively). Greater stress from COVID-19 was associated with the amount of ST for educational/professional purposes (*P*=.05), change in total ST (*P*=.006), change in entertainment ST (*P*=.01), and change in ST for educational/professional purposes (*P*=.02). Higher Alcohol, Smoking, and Substance Involvement Screening Test (ASSIST) tobacco scores were associated with greater change in total ST (*P*=.004), and higher pack-years were associated with greater change in SM ST (*P*=.003). Higher alcohol scores (*P*=.004) and servings of alcohol per week (*P*=.003) were associated with greater change in entertainment ST. Quarantining did not negatively impact these variables.

**Conclusions:**

There is no doubt ST and worsening mental health increased during COVID-19 in young adults. However, these findings indicate there are many significant associations between ST use and mental health. These associations are more complex than originally thought, especially since we found quarantining is not associated with mental health. Although other factors need to be further investigated, this study emphasizes different types of ST and degree of change in ST affect various groups of people in discrete ways. Acknowledging these findings can help young adults optimize their mental health during pandemics.

## Introduction

### Background

COVID-19, a novel disease, wreaked havoc on the world and caused an international public health emergency. Thus, quarantine and social distancing measures were implemented to decrease viral transmission and, in turn, resulted in dire consequences, including posttraumatic stress, fear, and anger [1,2]. These self-isolation measures caused work and education to move online, naturally increasing screen time (ST) use among groups [3]. Because ST was a controversial and well-studied topic prior to the pandemic, the interest revolving around ST and ST behaviors and the correlations they may have with various wellness variables became a popular target for mental health and public health researchers. Since the inception of the worldwide pandemic, many studies have demonstrated the harmful effects of ST on different population groups, particularly from mental and physical health perspectives [3]. Increased ST use during the pandemic has been linked to addictive behaviors, like alcohol, smoking, and sugar intake in adults [4].

It is obvious that ST use during the pandemic and the relationship to mental health are quite complex and multifactorial. For example, researchers have found shocking findings regarding ST in adolescents, pinpointing various demographic disparities and acknowledging mixed findings of ST depending upon modalities (ie, smartphone vs television), signifying that device ST may be an important factor to consider [5]. These findings indicate that the focus on ST should not solely be on time spent doing a particular activity on the screen, but it is important to further quantify activities in a way that might be meaningful to a person’s overall wellness. For instance, there has been a significant increase in online gaming during the pandemic associated with the need for individuals to socially connect due to stay-at-home mandates, which can either be beneficial or detrimental to a person’s well-being [6]. Understanding these complex associations can not only help improve future generations but also provide insight on novel treatment systems to potentially help others in the future during similar situations.

ST use during the COVID-19 pandemic largely focuses on its effects in children and adolescents, and there is little research on the young adult population (ages 18-30 years), which is of particular interest as this is the age of people attending college or university, developing their careers, and connecting with peers. Furthermore, specific ST use (ie, ST for studying vs ST for social media [SM] use) has not been looked at during the COVID-19 pandemic, and most of the focus is on total ST, as opposed to evaluating ST spent on specific activities without focusing on modality. Lastly, there is little analysis comparing the current use of ST to before the COVID-19 pandemic, as the degree of change could potentially hold vital insights and add information upon the complex relationship with ST and psychological state.

### Aim of This Study

Due to the emergence of the impact of specified ST use during the COVID-19 pandemic on declining mental health in younger generations around the world, this study originally aimed to collect information on ST patterns in young adults ages 18 years to 28 years, as well as additional wellness measures, like mental health, substance use, and overall well-being. The study not only collected data on 3 different ST uses (entertainment, SM, and educational or professional uses) but also aimed to assess ST changes between the pre-COVID-19 era and ongoing COVID-19 pandemic to expand the literature on ST patterns amid the pandemic.

## Methods

### Study Design and Sample Size

Data were collected via an online, international, cross-sectional, observational study that was conducted using the SurveyMonkey online survey platform (SurveyMonkey, San Mateo, CA) from September 2020 to January 2021. The research utilized convenience sampling to recruit participants in the targeted age range of 18 years to 28 years from the group’s medical institution in Saint Vincent and the Grenadines via word of mouth and school-wide emails. The study was publicized on popular SM websites (ie, Facebook, Twitter, LinkedIn) and research platforms (ie, SurveyCircle and SurveySwap) to collect additional responses on an international scale. The collection of responses was generated positively by the local campus (11/174, 6.3%), SM (55/174, 31.6%), and online research participant platforms (108/174, 62.1%).

A total of 294 respondents completed the questionnaire. Inclusion and exclusion criteria were added to validate all collected responses due to the structure of the research study. The inclusion criteria required all participants to be between the ages of 18 years and 28 years at the time of survey administration, which was verified by birth date. If a participant was outside of this age group, they were excluded from data analysis. Three validated questions were implemented within the survey to minimize random selection of choices. Respondents who incorrectly answered questions such as “Click to continue the survey” and “Please select agree/disagree for this answer” were excluded from data analysis. A total of 183 responses were validated, where 35 respondents were ineligible because they were out of the age range (ie, less than 18 years of age or greater than 28 years of age) and 76 respondents did not correctly answer the validation questions.

### Ethics Approval

An online consent form was presented to every participant to confirm their voluntary participation, allowing them to withdraw at any point or skip or refuse any questions they felt uncomfortable answering. All participants included in the study participated in the informed consent process with an additional informed consent for all individual participants for whom identifying information is included in this article. Participants did not receive any risks or reimbursements for their participation except the benefit of allowing this research group to gain more information on how mass pandemics can affect an individual’s mental health, which might help public health expand mental health options in the future. For the mental health section, we included an international mental health number and website the participant could go to should they experience distress from answering any of the questions. The research was approved by the Institutional Research Committee of the Saint James School of Medicine Saint Vincent and the Grenadines campus (Research Study #119). All procedures performed in studies involving human participants were per the ethical standards of the institutional or national research committee and with the 1964 Helsinki declaration and its later amendments or comparable ethical standards.

### Research Instruments

The pre-COVID-19 period was from October 2019 to December 2019, while the current COVID-19 period was from March 2020 to the day the participant participated in the survey. All questionnaires were tailored to specify assessment during the COVID-19 pandemic.

#### Social Demographics

The first section of the questionnaire collected general demographic information, like gender, age in 2020, ethnicity, race, employment status, country of residency in the past 6 months, income, and student status. Those who were students and not employed selected “unemployed, not looking for work.” Due to low sample sizes, the country of residence was categorized into continents during data analysis.

#### COVID-19–Related Questions

Participants were also asked a series of COVID-19 medically related questions. They answered questions (“Yes,” “No,” “Don’t know/unsure,” or “Refuse to answer”) regarding whether they had been tested for the virus and whether they were infected by SARS-CoV-2. Participants also answered questions regarding whether they quarantined and, if they quarantined, to specify if the quarantine time was past or current. Lastly, if they did quarantine (past or present), they were required to include the number of days they quarantined.

#### Screen Time Data

A validated questionnaire quantified the use of ST but was modified by asking participants for average ST use (hours/week) in the past 7 days during the COVID-19 pandemic for a particular category (entertainment, SM, and educational or professional purposes) [7]. “Thinking of an average week BEFORE the COVID-19 pandemic (October 2019-December 2019) and DURING (March 2020-Present Day), how much time do you spend using each of the following types of screens as the primary activity?” ST for the pre-COVID-19 era was retrospectively collected. Each category had examples listed. Entertainment included streaming websites, television, movies, music, and video games. SM included all major SM networks (ie, Facebook, Twitter, Snapchat) and related activities (eg, chatting, sharing information or pictures). Educational and professional ST included online lectures, webinars, business meetings, and video tutorials. If no time was spent, respondents were instructed to use “0” as their answer.

#### Mental Health (Depression, Anxiety, Psychological Impact of COVID-19, Substance Use, and Fear of COVID-19)

We used the Patient Health Questionnaire (PHQ-9) to assess depression, scoring each of the 9 DSM-IV criteria on a 4-point Likert-scale ranging from “not at all” (0), “several days” (1), “more than half the days” (2), and “nearly every day” (3), with a total sum ranging from 0 to 27 and scores equal to or greater than 10 indicating possible depression [8-10].

We used the Generalized Anxiety Disorder 7-item (GAD-7) to assess anxiety on a 4-point Likert scale from “not at all” (0) to “nearly every day” (3), where anxiety symptoms are classified as minimum (0-4), mild to moderate (5-14), and severe (15-21) [11,12].

The Impact of Events Scale-Revised (IES-R) is a 22-item measure, where scores over 24 indicate potential posttraumatic stress disorder (PTSD) [13-16]. The assessment for COVID-19 distress required calculating the overall IES-R scores, consisting of 22 items that include 7 items for intrusion, 8 for avoidance, and 7 for hyperarousal. The amount of difficulty experienced for each item is scaled as not at all “0,” a little bit “1,” moderately “2,” quite a bit “3,” and extremely “4.” Overall scores can point to a potential diagnosis of PTSD based on the range. A score from 24 to 32 indicates partial PTSD symptoms, a score from 33 to 38 indicates a probable diagnosis of PTSD, and a score above 39 suggests suppression of the immune system’s functioning. The means of each subset (intrusion, avoidance, hyperarousal) were also computed and used in the data analysis.

Illicit substance use was assessed using the World Health Organization’s (WHO’s) Alcohol, Smoking, and Substance Involvement Screening Test (ASSIST V3.0), which was modified to focus only on tobacco, alcohol, cannabis, amphetamine, and opioid use. The responses to each question are rated on a 5-point scale that ranges from “never,” “once or twice,” “monthly,” “weekly,” to “daily or almost daily.” Substance scores under 3 indicate no treatment needed, scores from 4 to 26 require brief treatment, and scores of 27 and higher require intensive treatment [17]. Additional questions evaluated pack-years and the average amount of alcohol consumed per week, with a guideline for serving size of alcohol (1 can/glass of beer = 1 glass of wine = 1 shot of liquor [eg, rum, tequila, vodka]). Due to low sample sizes, cocaine, amphetamine, and opioid ASSIST scores were dropped from the data analysis.

We used the Fear of COVID-19 Scale (FCV-19S) to assess an individual’s stress, anxiety, and fear over the virus. The questionnaire uses the Likert scale, ranging from 1 (strongly disagree) to 5 (strongly agree), to calculate the total score, with an overall sum score from 7 to 35: the higher the score, the greater the level of fear of COVID-19 [18].

#### Overall Well-being

The WHO's Five Well-Being Index (WHO-5) was used to measure subjective well-being, where 0 signifies the lowest quality of well-being and 100 indicates the highest quality of well-being [19]. Like the ST questions, these questions were asked twice—before and during the COVID-19 pandemic.

#### BMI

Each respondent was asked for their current height and their specific weight before and during the pandemic, along with dates of the weight recordings. The survey accepted each respondent's weight and height depending on whether they chose the US measurement system (pounds and feet/inches) or the universal metric system (kilograms and centimeters).

#### Sleep Quality

The Pittsburgh Sleep Quality Index (PSQI) was used to assess sleep quality in the past month (ie, during the COVID-19 pandemic) [20]. The sum score for each of the 7 subareas (subjective quality, sleep latency, sleep duration, habitual sleep efficiency, sleep disturbances, sleep medication use, and daytime dysfunction) yields a total score, which can be used to categorize sleep quality. Scores less than 5 indicate “good” sleep quality, whereas scores 5 or more indicate “poor” sleep quality.

### Data Analysis

Data analysis was conducted at the 95% significance level with SPSS version 25.0 (IBM Corp, Armonk, NY). Data analysis included paired and independent *t* tests, Levene test for equality of variances, Pearson correlation, chi-square tests, ANOVA tests, and Tukey post-hoc tests. Missing data and those who answered with “don't know” or “refuse to answer” were removed, as were small sample size numbers (n<15), like in the case of ASSIST cocaine, amphetamine, and opioid scores. If a participant had missing data for a scored section (ie, GAD-7, IES-R), that participant did not receive a score.

The dependent variables for analysis included demographic information (age in 2020, gender, ethnicity, race, student status, employment status, and continent of residence), alcohol weekly servings, pack-years, FCV-19S scores, past and current WHO-5 scores, past and current BMI, change in BMI, sleep duration, PSQI scores, PHQ-9 scores, GAD-7 scores, IES-R total, IES-R intrusion, IES-R avoidance, IES-R hyperarousal scores, and ASSIST scores for tobacco, alcohol, and cannabis.

Most participants were female (n=128), White (n=142), not Hispanic/Latinx (n=128), and from Europe (n=124). The average age in 2020 for all 183 participants was 23.43 (SD 2.54) years. Of the participants, 58.2% (106/183) were unemployed, and most participants were students (162/183, 88.5%). Over 70% (98/136, 72.1%) reported an income of less than US $50,000. Income was not associated with any of the dependent variables.

## Results

### Demographic Information

A comprehensive summary of demographics is in Table 1.

**Table 1:** Summary of demographic characteristics (n=183).

Individual-level variables			Results	
Age in 2020 (years), mean (SD)			23.43 (2.54)	
**Gender, n (%)**				
	Male	52 (28.9)		
	Female	128 (69.9)		
**Race, n (%)**				
	Caucasian/White	142 (77.6)		
	Black/African American	3 (1.6)		
	Asian	25 (13.7)		
	American Indian	1 (0.5)		
	Mixed	7 (3.8)		
**Ethnicity, n (%)**				
	Hispanic/Latinx	52 (28.9)		
	Not Hispanic/Latinx	128 (71.1)		
**Continent, n (%)**				
	Europe	124 (67.8)		
	North America	42 (23.0)		
	Asia	11 (6.0)		
	Africa	4 (2.2)		
	Australia	2 (1.1)		
**Employment status, n (%)**				
	Full-time	24 (13.2)		
	Part-time	47 (25.8)		
	Currently unemployed/student	106 (58.2)		
	Other	5 (2.7)		
**Income (US $), n (%)**				
	<50,000	98 (72.1)		
	50,000-99,999	22 (16.2)		
	100,000-149,999	8 (5.9)		
	≥150,000	8 (5.9)		
**Student status, n (%)**				
	Current student	162 (89.0)		
	Not a student	20 (11.0)		

### COVID-19–Specific Statistics and Associations

A breakdown of statistics on COVID-19 testing, diagnosis, and quarantine is provided in Table 2.

Only 40.3% (74/183) of the sample took a COVID-19 test, which was not associated with any of dependent variables. Of the participants, 6.7% (12/183) were diagnosed with a COVID-19 infection, which was only associated with ASSIST cannabis scores (*t*_33_=2.299, *P*=.004), 9.3% (17/183) were currently quarantining with an average quarantine time of 15.18 (SD 23.50) days, and 33.9% (62/183) quarantined in the past with an average quarantine time of 20.07 (SD 20.87) days. The Student *t* test analysis did not detect differences between groups quarantining or not quarantining for any of the dependent variables. The Pearson correlation analysis did not detect significant correlations between length of past quarantine and the dependent variables. However, the Pearson correlation did detect a positive correlation between length of current quarantine and current WHO-5 well-being scores (*r*_17_=0.531, *P*=.03) and past WHO-5 well-being scores (*r*_17_=0.626, *P*=.007).

**Table 2:** Summary of COVID-19 statistics (n=183).

Category	Results
Took a COVID-19 test^a^, n (%)	74 (40.4)
Had COVID-19^b^, n (%)	12 (6.7)
Currently in quarantine^a^, n (%)	17 (9.3)
Length of current quarantine (days)^c^, mean (SD)	15.18 (23.50)
Quarantined in the past^a^, n (%)	62 (33.9)
Length of quarantine in the past (days)^d^, mean (SD)	20.07 (20.87)

^a^n=183.

^b^n=180.

^c^n=17.

^d^n=61.

### Summary of ST Statistics

The average use of ST before and during COVID-19 for all domains is provided in Table 3.

**Table 3:** Comparison of screen time (ST) before and during the COVID-19 pandemic.

Type of ST	Before the COVID-19 pandemic	During the COVID-19 pandemic	*t* statistic (*df*)	*P* value
Total ST (hours/week), mean (SD)	14.26 (11.24)	23.26 (16.19)	–12.08 (178)	<.001
Entertainment ST (hours/week), mean (SD)	5.08 (4.85)	7.98 (6.45)	–10.11 (179)	<.001
Social media ST (hours/week), mean (SD)	4.49 (4.47)	6.79 (6.41)	–8.14 (179)	<.001
ST for educational or professional purposes (hours/week), mean (SD)	4.69 (5.54)	8.49 (7.05)	–8.63 (178)	<.001

### Before and During COVID-19 ST Comparisons

The paired *t* tests indicated that the mean ST for all domains significantly increased (*P*<.001; Table 3).

### Categorical Analysis of ST

For total ST during COVID-19, 20.7% (37/183) of participants used ST 0 hours to 10 hours per week, 38.0% (68/138) used ST 10 hours to 20 hours per week, and 41.3% (74/183) used ST at least 20 hours per week. There was a significant association with student status (*P*=.002; Table 4).

For entertainment ST during COVID-19, most participants (108/183, 60.0%) used ST 0 hours to 7 hours per week (Table 5). There was a significant association with student status (*P*=.02; Table 4), as well as between groups for PHQ-9 scores (*P*=.047; Table 6).

For SM ST during COVID-19, 70.0% (126/183) of participants used ST 0 hours to 7 hours per week (Table 5).

Regarding ST for educational or professional purposes during COVID-19, 54.7% (98/183) of participants used ST 0 hours to 7 hours per week, and 45.3% (81/183) used ST more than 7 hours per week (Table 5). There was a significant difference between groups for total IES-R scores (*P*=.05) and for IES-R intrusion scores (*P*=.004; Table 6).

**Table 4:** Significant associations between screen time (ST) data and demographic information.

Characteristics	Age in 2020			Gender			Ethnicity			Student status					Employment status			Continent of residence	
	Statistic	*P* value	Statistic		*P* value	Statistic		*P* value	Students, mean (SD)		Nonstudents, mean (SD)	Statistic	*P* value	Statistic		*P* value	Statistic		*P* value
Total ST (0-10, 10-20, >20 hours/week)	—^a^	—	—		—	—		—	—		—	*X*^2^_2,178_= 12.123	.002	—		—	—		—
Entertainment ST (0-7, >7 hours/week)	—	—	—		—	—		—	—		—	*X*^2^_2,179_= 5.958	.02	—		—	—		—
Change in total ST (hours)	—	—	—		—	—		—	—		—	*t*_176_= –2.774	.006	—		—	—		—
Change in entertainment ST (hours)	—	—	—		—	*F*_2,162_= 4.676		.01	3.10 (4.98)^b^		1.20 (1.61)^c^	*t*_177_= –3.960	<.001	—		—	—		—
Change in SM^d^ ST (hours)	—	—	—		—	—		—	2.55 (3.86)^b^		0.25 (2.40)^c^	*t*_177_= –2.594	.01	*F*_3,175_= 2.714		.046	—		—
Percent change in total ST	—	—	—		—	—		—	96.08 (172.15)^e^		16.49 (86.74)^c^	*t*_175_= –2.031	.04	—		—	—		—
Percent change in entertainment ST	—	—	—		—	*F*_2,152_= 4.127		.02	—		—	—	—	—		—	—		—
Percent change in SM ST	—	—	—		—	—		—	62.67 (81.75)^f^		8.08 (78.55)^c^	*t*_172_= –2.833	.005	*F*_3,170_= 3.192		.025	—		—
Percent change in educational or professional ST	—	—	—		—	—		—	163.18 (276.30)^g^		12.14 (121.97)^h^	*t*_141_= –2.019	.045	—		—	*F*_4,139_= 3.634		.008
Percent change in total ST (–200% to 0%, 0% to 100%, >100%)	—	—	—		—	—		—	—		—	*X*^2^_2,177_= 12.235	.002	—		—	—		—
Percent change in entertainment ST (–200% to 0%, 0% to 100%, >100%)	—	—	*X*^2^_2,167_= 8.462		.02	*X*^2^_4,155_= 17.371		.002	—		—	—	—	—		—	—		—
Percent SM ST (–200% to 0%, 0% to 100%, >100%)	*F*_2,172_= 3.744	.03	*X*^2^_2,172_= 6.694		.04	—		—	—		—	*X*^2^_2,174_= 7.155	.03	—		—	—		—

^a^Not applicable.

^b^n=159.

^c^n=200.

^d^SM: social media.

^e^n=157.

^f^n=154.

^g^n=129.

^h^n=14.

**Table 5:** Overview of categorical screen time (ST) during the COVID-19 pandemic (n=183).

Characteristics	0-7 hours/week, n (%)	>7 hours/week, n (%)
Entertainment	108 (60.0)	72 (40.0)
Social media	126 (70.0)	54 (30.0)
Educational or professional purposes	98 (54.7)	81 (45.3)

**Table 6:** Significant findings between screen time (ST) during the COVID-19 pandemic and secondary variables.

Characteristics	Entertainment ST					Educational or professional ST			
	0-7 hours/week	>7 hours/week	*F* statistic (*df*)	*P* value	0-7 hours/week		>7 hours/week	*F* statistic (*df*)	*P* value
PHQ-9^a^, mean (SD)	0.97 (0.62)^b^	1.19 (0.76)^c^	3.987 (1,166)	.047	—^d^		—	—	—
IES-R^e^ total, mean (SD)	—	—	—	—	17.58 (12.95)^f^		22.23 (15.96)^g^	3.918 (1,150)	.05
IES-R intrusion, mean (SD)	—	—	—	—	0.67 (0.59)^h^		0.99 (0.78)^i^	8.765 (1,163)	.004

^a^PHQ-9: Patient Health Questionnaire 9-item.

^b^n=101.

^c^n=67.

^d^Not applicable.

^e^IES-R: Impact of Events Scale-Revised.

^f^n=79.

^g^n=73.

^h^n=89.

^i^n=76.

### Average Change in ST

The calculation for the average ST change was the difference between the ST during and the ST before the COVID-19 pandemic.

For total ST, the average change in ST was 9.00 hours per week (SD 9.97, n=179; Table 7). There was a significant difference in student status (*P*=.002; Table 4). There were also correlations with WHO-5 scores before COVID-19 (*P*=.04), IES-R total (*P*=.006) and intrusion scores (*P*=.003), and tobacco scores (*P*=.03; Table 8).

For entertainment, the average change in ST (n=180) was 2.90 (SD 3.85) hours per week (Table 7). Students were significantly different from nonstudents (*P*<.001; Table 4). There were associations with WHO-5 scores before COVID-19 (*P*=.045); GAD-7 scores (*P*=.03); IES-R total (*P*=.01), intrusion (*P*=.02), and hyperarousal scores (*P*=.045); sleep duration (*P*=.03); PSQI scores (*P*=.01); alcohol servings per week (*P*=.004); and alcohol scores (*P*=.003; Table 8).

For SM, the average change in ST (n=180) was 2.29 (SD 3.78) hours per week (Table 7). There was a significant difference between groups for student status (*P*=.001) and employment status (*P*=.046; Table 4). The Tukey post-hoc analysis showed full-time employees (mean 0.22, SD 1.95; n=23) were significantly different than part-time employees (mean 2.68, SD 3.19; n=47; *P*=.05; 95% CI –4.93 to 0.0005) and unemployed individuals (mean 2.56, SD 4.20; n=104; *P*=.04; 95% CI –4.57 to –0.11). There was also a correlation with pack years (*P*=.007; Table 8).

For educational or professional uses, the average change in ST (n=179) was 3.80 (SD 5.89) hours per week (Table 7). There were correlations with IES-R total (*P*=.02) and intrusion (*P*=.02) scores (Table 8).

**Table 7:** Summary of change and percent change in screen time (ST) statistics.

Individual-level variables	Results, mean (SD)
Change in total ST (hours)	9.00 (9.97)^a^
Change in entertainment ST (hours)	2.90 (3.85)^b^
Change in SM ST (hours)	2.29 (3.78)^b^
Change in educational or professional ST (hours)	3.80 (5.89)^a^
Percent change in total ST (%)	88.28 (166.79)^c^
Percent change in entertainment ST (%)	93.29 (159.10)^d^
Percent change in SM ST (%)	56.94 (83.08)^e^
Percent change in educational or professional ST (%)	150.14 (268.59)^f^

^a^n=179.

^b^n=180.

^c^n=178.

^d^n=169

^e^n=175.

^f^n=144.

**Table 8:** Significant associations between change in screen time (ST) between the before COVID-19 and during COVID-19 periods and secondary variables.

Characteristics	Change in total ST (hours)			Change in entertainment ST (hours)			Change in SM^a^ ST (hours)			Change in educational or professional ST (hours)	
	Statistic	*P* value	Statistic		*P* value	Statistic		*P* value	Statistic		*P* value
WHO-5^b^ scores prior to COVID-19	*r*_179_=0.155	.04	*r*_180_=0.149		.045	—^c^		—	—		—
PSQI^d^	—	—	*r*_99_=0.256		.01	—		—	—		—
Sleep duration	—	—	*r*_180_=0.164		.03	—		—	—		—
GAD-7^e^	—	—	*r*_175_=0.169		.03	—		—	—		—
IES-R^f^ total	*r*_152_=0.221	.006	*r*_153_=0.204		.01	—		—	*r*_152_=0.185		.02
IES-R intrusion	*r*_165_=0.234	.003	*r*_166_=0.181		.02	—		—	*r*_165_=0.187		.02
IES-R hyperarousal	—	—	*r*_176_=0.151		.045	—		—	—		—
ASSIST^g^ tobacco scores	*r*_32_=0.387	.03	—		—	—		—	—		—
Pack-years (smoking)	—	—	—		—	*r*_33_=0.460		.007	—		—
ASSIST alcohol scores	—	—	*r*_114_=0.264		.004	—		—	—		—
Servings of alcohol per week	—	—	*r*_117_=0.269		.003	—		—	—		—

^a^SM: social media.

^b^WHO-5: World Health Organization Well-Being Index 5-item.

^c^Not applicable.

^d^PSQI: Pittsburg Sleep Quality Index.

^e^GAD-7: Generalized Anxiety Disorder 7-item.

^f^IES-R: Impact of Events Scale-Revised.

^g^ASSIST: Alcohol, Smoking, and Substance Involvement Screening Test.

### Average Percent Change in ST

The average percent change in ST was calculated by dividing the change number by the amount of ST before COVID-19 and multiplying it by 100.

The average percent change in total ST (n=178) was 88.28% (SD 166.79%; Table 7). Students had statistically significant higher percent changes in total ST than nonstudents (*P*=.04; Table 4). There were also correlations with WHO-5 past and current scores (*P*=.004 and *P*=.001, respectively; Table 9).

For entertainment, the average percent change in ST (n=169) was 93.29% (SD 159.10%; Table 7). There is a positive correlation with alcohol weekly servings (*P*<.001; Table 9).

The average percent change in SM ST (n=175) was 56.94% (SD 83.08%; Table 7). Students had statistically significant higher percent changes in SM ST than nonstudents (*P*=.005; Table 4). There was a significant difference between groups for employment status (*P*=.03; Table 4). The Tukey post-hoc analysis showed full-time employees were statistically significant from unemployed individuals (*P*=.049; 95% CI –97.85 to –0.20). There are correlations with GAD-7 scores (*P*=.007), PHQ-9 scores (*P*=.002), and IES-R intrusion scores (*P*=.02; Table 9).

For educational or professional uses, the average percent change in ST (n=144) was 150.14% (SD 268.59%; Table 7). Students had statistically significant higher percent changes in educational or professional ST than nonstudents (*P*=.045; Table 4). In addition, there was a significant difference between groups for continent of residence (*F*_4,139_=3.634, *P*=.008; Table 4). The Tukey post-hoc analysis showed those who lived in Europe (mean 154.35, SD 236.61; n=100; *P*=.01; 95% CI –794.30 to –63.67) and North America (mean 68.52, SD 183.52; n=30; *P*=.003; 95% CI –896.16 to –133.45) had less change than those in Africa (mean 583.33, SD 141.42; n=4).

**Table 9:** Significant associations between percent change in screen time (ST) between the before COVID-19 and during COVID-19 periods and secondary variables.

Characteristics	Percent change in total ST (%)			Percent change in entertainment ST (%)			Percent change in SM^a^ ST (%)	
	Statistic	*P* value	Statistic		*P* value	Statistic		*P* value
WHO-5^b^ scores prior to COVID-19	*r*_178_=0.217	.004	—^c^		—	—		—
WHO-5 scores during COVID-19	*r*_175_=0.191	.01	—		—	—		—
PHQ-9^d^	—	—	—		—	*r*_163_=0.212		.007
GAD-7^e^	—	—	—		—	*r*_170_=0.235		.002
IES-R^f^ intrusion	—	—	—		—	*r*_161_=0.184		.02
Servings of alcohol per week	—	—	*r*_112_=0.356		<.001	—		—

^a^SM: social media.

^b^WHO-5: World Health Organization Well-Being Index 5-item.

^c^Not applicable.

^d^PSQI: Pittsburg Sleep Quality Index.

^e^GAD-7: Generalized Anxiety Disorder 7-item.

^f^IES-R: Impact of Events Scale-Revised.

### Categorical Analysis of Percent Change in ST

For the percent change in total ST, less than one-quarter of participants (27/178, 15.2%) decreased ST use, but more than three-quarters (151/178, 84.0%) increased it (Table 10). There was a significant association with student status (*P*=.002; Table 4). There was a significant difference between groups for total IES-R scores (*P*=.01) and IES-R intrusion (*P*=.03; Table 11).

For percent change in entertainment, more than one-quarter of participants (44/169, 26.0%) decreased ST use, and almost three-quarters (125/169, 74.0%) increased it (Table 10). There was a significant association with gender (*P*=.02; Table 4). There was a significant difference between groups for WHO-5 scores during the pandemic (*P*=.01), GAD-7 scores (*P*=.047), IES-R total scores (*P*=.03), IES-R intrusion scores (*P*=.01), and IES-R hyperarousal scores (*P*=.01; Table 12).

For percent change in SM ST, 29.7% (52/175) of participants decreased SM use, but over 70% (123/175, 70.3%) increased it (Table 10). There was a significant association with gender (*P*=.04), student status (*P*=.03), and average age in 2020 (*P*=.03; Table 4). For average age, the Tukey post-hoc analysis found the mean value for the –200% to 0% category (mean 23.01, SD 2.49 years; n=97) was significantly different from the mean value for the 0% to 100% category (mean 23.38, SD 2.16 years; n=26; *P*=.02; 95% CI –2.20 to –0.16). There was a significant difference between groups for PHQ-9 scores (*P*=.03) and IES-R total (*P*=.006), intrusion (*P*=.001), and avoidance (*P*=.02) scores (Table 13).

For percent change in ST for educational or professional purposes, 41.0% (59/183) of participants decreased ST for educational or professional purposes, and over one-half (124/183, 59.0%) increased (Table 10). There was a significant difference between groups for BMI during COVID-19 (*P*=.03; Table 14).

**Table 10:** Overview of percent change in screen time (ST) during the COVID-19 pandemic.

Characteristics	–200% to 0%, n (%)	0% to 100%, n (%)	>100%, n (%)
Total ST	27 (15.2)	96 (53.9)	55 (30.1)
Entertainment ST	44 (26.0)	86 (50.9)	39 (23.1)
Social media ST	52 (29.7)	97 (55.4)	26 (14.9)
ST for educational or professional purposes	59 (41.0)	36 (25.0)	49 (34.0)

**Table 11:** Significant associations between total percent change in screen time (ST) categories and secondary variables.

Characteristics	Percent change in total ST				*F* statistic (*df*)		*P* value
	–200% to 0%	0% to 100%	>100%				
IES-R^a^ total, mean (SD)	11.43 (9.97)^b^	20.45 (12.72)^c^	22.25 (18.04)^d^	4.370 (2,148)		.01	
IES-R intrusion, mean (SD)	0.48 (0.45)^e^	0.84 (0.66)^f^	0.92 (0.82)^g^	3.629 (2,161)		.03	

^a^IES-R: Impact of Events Scale-Revised.

^b^n=21.

^c^n=82

^d^n=48.

^e^n=25.

^f^n=87.

^g^n=52.

**Table 12:** Significant associations between percent change in entertainment screen time (ST) categories and secondary variables.

Characteristics	Percent change in entertainment ST				*F* statistic (*df*)		*P* value
	–200% to 0%	0% to 100%	>100%				
WHO-5^a^ scores during COVID-19, mean (SD)	48.93 (22.29)^b^	46.64 (18.45)^c^	36.53 (20.04)^d^	4.586 (2,163)		.01	
GAD-7^e^, mean (SD)	6.67 (5.03)^f^	8.40 (5.26)^g^	9.62 (5.93)^h^	3.122 (2,162)		.047	
IES-R^i^ total, mean (SD)	16.84 (11.06)^j^	19.28 (12.55)^k^	25.69 (20.52)^l^	3.486 (2,140)		.03	
IES-R intrusion, mean (SD)	0.63 (0.55)^m^	0.81 (0.62)^n^	1.10 (0.94)^o^	4.525 (2,152)		.01	
IES-R hyperarousal, mean (SD)	0.94 (0.75)^b^	0.93 (0.62)^c^	1.36 (0.94)^j^	4.737 (2,162)		.01	

^a^WHO-5: World Health Organization Well-Being Index 5-item.

^b^n=43.

^c^n=85.

^d^n=38.

^e^GAD-7: Generalized Anxiety Disorder 7-item.

^f^n=42.

^g^n=84.

^h^n=39.

^i^IES-R: Impact of Events Scale-Revised.

^j^n=37.

^k^n=74.

^l^n=32.

^m^n=40.

^n^n=78.

^o^n=35.

**Table 13:** Significant associations between percent change for social media (SM) screen time (ST) categories and secondary variables.

Characteristics	Percent change in SM ST				*F* statistic (*df*)		*P* value
	–200% to 0%	0% to 100%	>100%				
PHQ-9^a^, mean (SD)	8.35 (6.28)^b^	10.80 (5.43)^c^	11.70 (7.63)^d^	3.467 (2,160)		.03	
IES-R^e^ total, mean (SD)	14.17 (9.92)^f^	22.64 (13.82)^g^	20.36 (20.88)^h^	5.352 (2,146)		.006	
IES-R intrusion, mean (SD)	0.51 (0.46)^b^	0.95 (0.68)^i^	0.93 (0.98)^j^	7.083 (2,158)		.001	
IES-R avoidance, mean (SD)	0.70 (0.62)^k^	1.10 (0.79)^l^	1.01 (1.15)^d^	3.934 (2,157)		.02	

^a^PHQ-9: Patient Health Questionnaire 9-item.

^b^n=49.

^c^n=91.

^d^n=23.

^e^IES-R: Impact of Events Scale-Revised.

^f^n=46.

^g^n=81.

^h^n=22.

^i^n=88.

^j^n=24.

^k^n=48.

^l^n=89.

**Table 14:** Significant associations between percent change for educational or professional screen time (ST) categories and secondary variables.

Characteristics	Percent change in educational/professional ST				*F* statistic (*df*)		*P* value
	–200% to 0%	0% to 100%	>100%				
BMI during COVID-19, mean (SD)	24.73 (6.74)^a^	23.00 (3.53)^b^	22.18 (3.49)^c^	3.517 (2,141)		.03	

^a^n=59.

^b^n=36.

^c^n=40.

## Discussion

### Main Findings

Overall, our findings corroborate with those of other studies indicating that ST increased significantly during COVID-19 in all domains compared with before COVID-19 (all *P*<.001; Table 2) [3,5,21]. Since most of our sample consisted of students, it is expected that the change and percent change in ST for educational and professional purposes had the greatest increase (Table 7). Yet, we found the percent change in ST for SM increased by around 57% compared with entertainment, at 93% (Table 7), indicating our sample drastically increased their time spent on entertainment and only somewhat increased time spent on SM. Higher ST levels for SM were associated with higher social connectedness in high school students, which would be expected during quarantine situations in students, as they might use SM networks to keep in touch with friends during remote schooling [22]. Discovering that entertainment had a higher increase in ST sheds light onto the patterns of young adults who are primarily students during the pandemic and how this population sought more time on entertainment and less on social networking. Even evaluating categorical breakdowns, more participants spent ≥7 hours per week (72/180, 40.0%) on entertainment than SM (54/180, 30.0%; Table 5), and more participants increased their ST time ≥100% for entertainment compared with before the pandemic (39/169, 23.1%) than for SM (26/175, 14.9%; Table 10). This could potentially be explained by the fact that researchers are now finding that playing video games, which was included as an example of entertainment ST, is now a new form of social contact for people during COVID-19 and could even have a positive impact on people, which may indicate why quarantine was not associated with negative mental health findings [23].

The differences between SM and entertainment ST variables are stark and emphasize discrete characteristics. For one, we found that there were no significant findings for the dependent variables and SM ST between the 0-7 hours/week and ≥7 hours/week groups, but there were differences between the groups for entertainment ST and depression scores (*P*=.047; Table 6). Furthermore, greater change in SM ST compared with before the pandemic was only associated with higher pack-years (*P*=.007), but greater changes in entertainment were associated with higher well-being scores (*P*=.045), poorer sleep quality *(P*=.01), longer sleep duration (*P*=.03), more severe anxiety (*P*=.03), greater stress from the pandemic (*P*=.02), higher levels of alcohol abuse (*P*=.004), and more servings of alcohol per week (*P*=.003; Table 8). However, the greater degree of change compared with before the pandemic is critical, as the greater the percent change in SM has associations with more severe depression (*P*=.007), more severe anxiety (*P*=.002), and more stress from the pandemic (*P*=.02), whereas a greater degree of change in entertainment ST was only associated with higher servings of alcohol per week (*P*<.001; Table 9). Once these degrees of change are broken down into decreased percent change, mild to moderate percent change (0%-100% compared with before), and extreme percent change (≥100% compared with before), more correlations become apparent and reiterate that degree of change compared with before does play a role in areas of well-being, anxiety, depression, and stress from the pandemic (Tables 12 and 13). These findings suggest a complex relationship between type of ST and increase compared with before, especially since this study found that total ST did not have any associations with the dependent variables outside of gender.

Current research emphasizes that quarantine affects a person’s mental health, yet we did not find that in our sample size [24]. However, a recent meta-analysis did suggest that quarantine affects varying groups of people differently, and our population might possess a quality or multiple qualities that affects them less than others [25]. A study in Ecuador did mention that, although students express discontent in self-isolation, many students are happy with remote learning, and only 16% have clinical depression [26]. Since our sample is primarily students, this also might be a reason for these findings, especially since we found that, if people were quarantining at the time of taking the survey, longer time spent quarantining was associated with higher well-being scores (*P*=.03). Another reason behind this could be due to increased levels of entertainment ST, and despite finding associations with negative mental health consequences, gaming can be a positive impact. Future studies need to assess ST, entertainment ST, gaming-specific ST, and types of games played with these factors to provide more concrete explanations [23]. In addition, although a COVID-19 diagnosis was not found to be associated with mental health or wellness in this sample, we did find that there was an association with ASSIST cannabis scores (*P*=.004), potentially indicating an association between COVID-19 diagnosis and cannabis use, which could be explained by the fact that researchers postulate that regular cannabis users may be more vulnerable to COVID-19 infections [27].

Although our study did show some demographic differences, the most significant demographic grouping difference was seen between students and nonstudents. There were significant differences (*P*<.05) for all but 2 ST characteristics—percent change in total ST and categorical analysis percent change for entertainment ST (Table 4). Although it is well-known that students have increased ST during the pandemic due to remote learning [28], a noteworthy finding is that students were more likely to have a higher degree of percent change for entertainment and SM ST compared with nonstudents. This further reiterates the point that total ST only provides minimal information and detailing the type of ST and the amount spent will provide greater insights into ST behaviors and patterns. Aside from students, we also found that full-time employees were less likely to have a drastic change in ST for SM and average percent change in SM than part-time employees or unemployed individuals (*P*=.046 and *P*=.03, respectively; Table 4), indicating employment may play a role in the amount of SM usage during a pandemic. A similar finding was recently found, associating it with increased sedentary time [29]. Furthermore, percent change in SM ST may have a generational link, as those who had a general percent decrease in SM were younger than those who had an increase (*P*=.03; Table 4). Although we did not find any differences between races in our sample, we did find differences between ethnicities for change in entertainment ST (*P*=.01) and percent change in entertainment ST (*P*=.02; Table 4), which has been postulated to be due to structural and systemic racism-driven factors proposed by a recently published study [5]. These discrepancies encourage race and ethnicity-specific studies to learn more about these patterns to help benefit these minority populations who have largely struggled during the COVID-19 pandemic. Lastly, one of the more unique findings is the difference between groups for the continent of residence for the average percent change in ST for educational and professional purposes (*P*=.001; Table 4). On average, those who lived in Africa had a more remarkable change than the participants who lived in Europe or North America. This finding implies participants who live in Africa drastically increased their time devoted to education and profession during the pandemic than before the pandemic. This discrepancy can be due to multiple factors, from cultural to structural ones.

There was substantial evidence linking ST to multiple adverse wellness factors, especially with psychological distress from the pandemic. IES-R total scores were more significant for those who spent ≥7 hours per week on ST for educational or professional reasons (*P*=.05; Table 6) but not for other ST variables. This unique finding suggests a correlation between educational or professional ST and psychological distress. Furthermore, like studies before, we found an association between depression and entertainment ST (*P*=.047; Table 6) [30], not to mention the various post-hoc tests for percent change and IES-R indicate how the IES-R scores were notably increased when the percent change in ST increased; this was seen for all but educational or professional ST. It is critical to note depression scores were not correlated with total ST use, indicating certain types of ST do not influence depression, but others, like entertainment, might. In addition, we found associations with anxiety and average change in entertainment ST (*P*=.03; Table 8), something sparse in current literature. This finding is novel because, like depression, it is not linked with total ST but rather a specific type of ST.

Since the literature is lacking in information about substance use and ST, our findings add insight into multiple substances. For one, we reiterated the link between increased entertainment ST and alcohol drinking during COVID-19 [4]. We also found ASSIST tobacco scores increased with greater change in total ST use, similar to other studies’ scores (*P*=.03; Table 8) [31]. This could be due to stress, as increased academic stressors can increase smoking behaviors [32]. Our research also found links between the number of pack-years and change in SM ST (*P*=.007; Table 8), the first mentioned in current literature.

We did not find many associations between sleep quality and ST despite literature indicating otherwise [33,34]. Physiotherapy students with excessive ST had poor sleep quality, yet our data suggest this is only the case for a significant change in ST for entertainment (*P*=.01; Table 8) [33]. We also expected to find significant associations with ST and BMI [35] but found those who decreased their ST had a significantly higher BMI than those who increased their ST (*P*=.03; Table 14). Aside from this association, there were no significant findings regarding BMI, indicating ST was not an impactful factor on BMI scores during COVID-19 in our sample.

### Limitations

This study has multiple limitations. Some variables had small sample sizes and needed to be removed from the data analysis. The study was conducted online, and participants were recruited online, thus providing an amount of bias in reporting since these participants may have more ST than others. We were unable to stratify by country due to low sample sizes; thus, we grouped participants into continents, which generalizes and takes away from the discrete culture within one country. For one, we did not collect information on psychiatric history. In addition, calculating ST was focused on hours per week and did not scientifically measure time spent on each device, nor did we collect information on modalities. Furthermore, the inability to recall could influence and provide a degree of bias in the pre-COVID-19 variables. Lastly, our population did not properly mimic the overall young adult population, as most were White, students, and female.

### Conclusion

This study is the first in the current literature to focus on ST behaviors and patterns during the COVID-19 pandemic in young adults and to identify various correlations in ST and demographics, mental health, substance use, and wellness factors. Our findings show that total ST alone is not enough to predict mental health of young adults during the pandemic and that certain types of ST, in particular for entertainment and SM, provide more insight into psychological health and other wellness factors. Furthermore, the amount and degree of change of ST compared with before the pandemic are more telling of psychological impact and wellness. These insights provide a baseline for future researchers to continue to explore these untouched depths in hopes to find personalized solutions to benefit young adults and their mental health and wellness in the future with similar pandemics.

## Abbreviations

ASSIST: Alcohol, Smoking, and Substance Involvement Screening Test
FCV-19S: Fear of COVID-19 Scale
GAD-7: Generalized Anxiety Disorder 7-item
IES-R: Impact of Events Scale-Revised
PHQ-9: Patient Health Questionnaire 9-item
PSQI: Pittsburg Sleep Quality Index
PTSD: posttraumatic stress disorder
SM: social media
ST: screen time
WHO: World Health Organization
WHO-5: World Health Organization Well-Being Index 5-item

## Data Availability

The raw data presented in this study are not publicly available, but senior author MTW can provide additional statistical analysis in response to a reasonable request. Participants received assurance that all responses would remain confidential within the research team with limited access to databases except for the listed co-authors.
